# Risk assessment of sexual behaviors in serodiscordant couples

**DOI:** 10.1186/1471-2334-13-S1-P64

**Published:** 2013-12-16

**Authors:** Maria Alexandra Largu, Carmen Manciuc, Liviu Jany Prisăcariu, Cristina Nicolau, Daniela Stoica, Carmen Dorobăț

**Affiliations:** 1 Infectious Diseases Hospital “Sf Parascheva”, Iaşi, Romania

## Background

This paper aims to assess the risk of sexual behaviors in serodiscordant couples in the Infectious Diseases Hospital “Sf Parascheva”, Iaşi, Romania.

## Methods

We assessed 40 persons from serodiscordant couples, in the hospital’s psycho-social assistance office. Of these, 20 were HIV-positive young men and women registered in the Regional HIV/AIDS Center. Assessments were made between January and August 2013. The instrument used was a questionnaire developed by the psychologist, with questions relating to the frequency of sexual intercourse and contraceptive methods used.

## Results

All the young people we assessed were part of heterosexual couples, with equal numbers of males and females. The average age was 24.76 years, with a minimum of 18 and maximum of 34 years. Participants were mostly post-secondary education or training (31%), followed in proportion by secondary education (grades 4-8, 21%), but with a high percentage of high school and university (20%) as well. Thirty participants belonged to a family, while 10 were from foster care or placement centers. 46% of participants were in a relationship based on dating without living together, and equal proportions of 27.5% were either married or cohabiting. Regarding the duration of the relationship in which they were involved, the majority of participants (51%) had an ongoing relationship for more than one year, 30% between 6 months and one year, 15% between 3 and 6 months and only 3.8% estimated the duration of their relationship for less than 3 months. Eighty percent declared they have sex once a week or more, 5% less often than once a week, 10% said they did not have sex, and the remaining 5% refused to answer the question. Of the 85% of sexually active, 29.4% reported using a condom every time, 58.8% sometimes and 11.7% never.

## Conclusion

HIV-positive patients and their HIV-negative partners still assume considerable risk and consciously ignore protection against HIV transmission. However, the results of this study are up for debate due to a certain attitude regarding the disclosure of personal details about one’s sex life, still common in the current social context.

